# Combination testing confirming clindamycin induced acute generalized exanthematous pustulosis

**DOI:** 10.1016/j.jdcr.2025.06.005

**Published:** 2025-06-18

**Authors:** Vinh-An Vo, Elizabeth Dupuy, Christine R.F. Rukasin

**Affiliations:** a Department of Pediatrics, Phoenix Children’s, Phoenix, Arizona; b Division of Dermatology, Phoenix Children’s, Phoenix, Arizona; c Division of Allergy and Immunology, Phoenix Children’s, Phoenix, Arizona; d Division of Allergy, Asthma and Clinical Immunology, Mayo Clinic, Phoenix and Scottsdale, Arizona

**Keywords:** acute generalized exanthematous pustulosis, AGEP, clindamycin, intradermal testing, patch testing, severe cutaneous adverse reaction

## Introduction

Acute generalized exanthematous pustulosis (AGEP) is a severe cutaneous adverse reaction often caused by drug exposure such as antibiotics; however, other triggers include infections, vaccinations, and even spider bites.^1^ The typical cutaneous manifestation is rapid development of sterile, nonfollicular pustules on an edematous and erythematous base followed by desquamation.^1^ Systemic symptoms such as fever and leukocytosis are often present.^1^ Discontinuation of the offending agent leads to resolution and treatment is supportive often with topical corticosteroids. We report a case of AGEP with exposure to multiple medications and the use of patch and intradermal testing to confirm clindamycin as the culprit. The patient consented to sharing information case details and images.

## Case report

**Fig 1:**
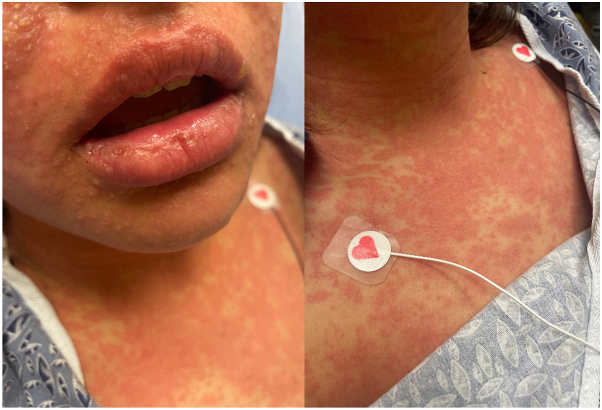
Acute generalized erythematous pustulosis. Numerous monomorphic pustules on an erythematous base with associated facial swelling and diffuse edematous and erythematous thin plaques with superimposed small monomorphic pustules on the chest and neck.

A previously healthy 18-year-old female presented to the emergency room for erythema, swelling, and pain of the left earlobe after wearing new earrings. Due to severity and location, she underwent a computerized tomography scan with intravenous contrast, iohexol, was treated with ceftriaxone, and discharged with oral clindamycin and naproxen (Table I). The following day, a widespread morbilliform rash erupted prompting evaluation by a dermatologist who prescribed dicloxacillin, topical clindamycin, and microdacyn spray. The next day, the patient developed a fever to 38.4°C and the eruption worsened with an appearance of pink macules and pustules along the face and neck, large edematous pink plaques with tiny pustules on the bilateral axilla, chest, abdomen, scattered pink edematous macules on the lower extremities (Fig 1). Laboratory evaluation demonstrated a complete blood count with differential within normal range and normal procalcitonin level. AGEP was suspected due to clinical features, but no biopsy was performed. Treatment included oral prednisone, topical corticosteroids, and all other medications were stopped. The rash completely resolved within 1 month. As the patient had received multiple medications in the 48 hours prior to eruption, the specific culprit was difficult to determine. Therefore, she was instructed to avoid contrast, nonsteroidal anti-inflammatory drugs, penicillins, clindamycin, and ceftriaxone.

**Table I:** Timeline of drug exposures, symptom development, and evaluation findings

Timing	Exposures	Symptoms
Index reaction		
Day 0	Iohexol Ceftriaxone Clindamycin Naproxen	Earlobe cellulitis–erythema, swelling, pain
Day 1	Dicloxacillin Topical clindamycin Topical microdacyn	Widespread morbiliform eruption
Day 2	Prednisone Topical corticosteroids	AGEP–fever, large edematous pink patches with pustules
Testing results Day 1 (24 h)-Intradermal testing	ampicillin 25 mg/ml ceftriaxone 10 mg/ml iohexol 30 mg/ml iodixanol 32 mg/ml clindamycin 15 mg/ml	Erythema, induration, and papular eruption at the intradermal site of clindamycin 15 mg/ml
Day 2 (48 h)- Intradermal testing	ampicillin 25 mg/ml ceftriaxone 10 mg/ml iohexol 30 mg/ml iodixanol 32 mg/ml clindamycin 15 mg/ml	Worsening erythema, induration, and tiny pustules at the intradermal site of clindamycin 15 mg/ml
Day 3 (72 h) - Intradermal testing	ampicillin 25 mg/ml ceftriaxone 10 mg/ml iohexol 30 mg/ml iodixanol 32 mg/ml clindamycin 15 mg/ml	Worsening erythema, induration, and tiny pustules at the intradermal site of clindamycin 15 mg/ml
Day 4 (96 h)-Patch testing interpretation	amoxicillin 10% ampicillin 5% cefpodoxime 10% aspirin 10% naproxen 10% ibuprofen 5% and 10% clindamycin 10% dicloxacillin 10%	Erythema and induration at the patch site for clindamycin 5%

**Fig 2:**
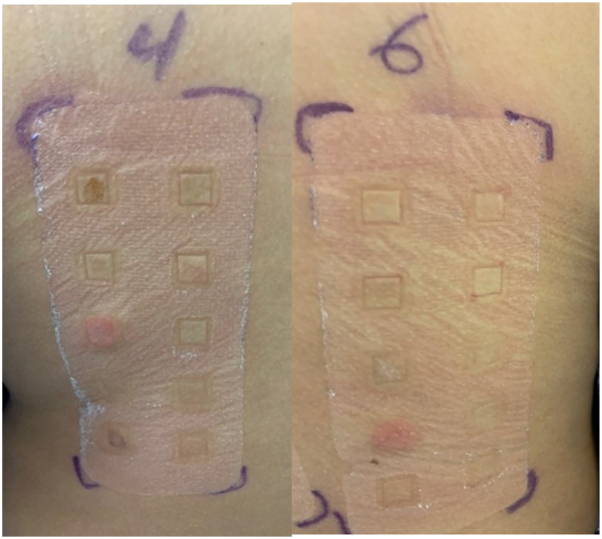
Patch testing results. Panel 4: metal testing, positive is nickel with erythema 2+. Panel 6: drug testing, positive to clindamycin with erythema and induration 2+.

**Fig 3:**
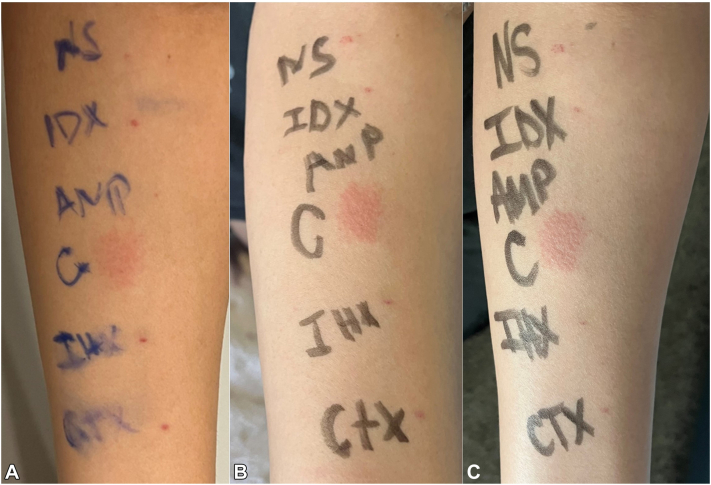
Intradermal testing results. Intradermal testing positive to clindamycin with erythematous wheal and papules. **A,** picture at 24 h, **B,** picture at 48 h, and **C,** picture at 72 h. *AMP*, Ampicillin; *C*, clindamycin; *CTX*, ceftriaxone; *IDX*, iodixanol; *IHX*, iohexol; *NS*, normal saline.

The patient was referred for drug allergy evaluation which included patch testing (PT) and intradermal testing (IDT) with delayed interpretation. The patient completed patch testing to amoxicillin 10%, ampicillin 5%, cefpodoxime 10%, aspirin 10%, naproxen 10%, ibuprofen 5% and 10%, clindamycin 10%, dicloxacillin 10%. Intradermal testing to ampicillin 25 mg/ml, ceftriaxone 10 mg/ml, iohexol 30 mg/ml, iodixanol 32 mg/ml, and clindamycin 15 mg/ml were completed with delayed interpretation after 24 hours. Patch testing was positive at 96 hours to clindamycin (2+) (Table I). At 24 hours, the site of the IDT to clindamycin developed erythema, induration, and a papular rash which the patient reported was identical to the index eruption (Figs 2 and 3). To ensure safety of other medications, an oral amoxicillin challenge was completed without any symptoms.After discussion of risks/benefits and limitations of testing, all medications including nonsteroidal anti-inflammatory drugs, penicillins, contrast, and cephalosporins were deemed safe to use. The patient was instructed to avoid clindamycin.

## Discussion

AGEP, although rare, is a severe cutaneous adverse reaction and can be due to drugs, infection, or vaccines.^2^ In patients who receive multiple medications within 48 hours of eruption, identification of a culprit may be difficult on history alone. Methods for drug allergy testing include skin prick testing, intradermal testing with immediate or delayed interpretation, patch testing, and re-exposure challenge.^3^ The evaluation strategy is guided by a thorough history and phenotyping of the index reaction. For immediate reactions, such as anaphylaxis, skin prick testing and IDT can be used. For delayed hypersensitivity reactions that are T-cell mediated, such as AGEP and drug related eosinophilia and systemic symptoms, PT and IDT, can be utilized to potentially identify the culprit drug(s).For immediate or delayed reactions that do not seem consistent with an immune mediated reaction, if skin testing is not available, or to confirm a negative skin or patch test, re-challenge is frequently utilized. IDT involves injection of a nonirritating concentration of a sterile drug under the skin. It can be used to assess IgE-mediated responses if interpreted 15-20 minutes after placement, but monitoring the site over the next 1-3 days will identify a Tcell response. The limitation of IDT is only sterile preparations of drugs can be used. Patch testing involves application of small amounts of suspected allergens diluted in an appropriate vehicle to the skin with adhesive for a minimum of 48 hours to identify a T-cell response. This can be completed with nonsterile and sterile preparations of medications. Re-challenge is administration of the medication or vaccine under observation to monitor for symptoms consistent with a hypersensitivity reaction.

The drug tested and type of cutaneous reaction are important factors in the diagnostic value of PT and delayed IDT.^4^ In AGEP, PT has up to 58% sensitivity for specific implicated medications^4^^,^^5^ with positive results for the most frequent agents associated with AGEP,^6^ defined as agents for which a 10% dilution in petroleum has resulted in positive PT for approximately 15% to 30% of patients.^7^ Limited data are available on delayed IDT, but the consensus is that it may be a potentially useful tool.^4^^,^^5^^,^^8^^,^^9^

In this case, the patient received multiple medications including ceftriaxone, intravenous contrast, clindamycin, and naproxen all of which are potential culprits. With polypharmacy and an unclear trigger, utilizing both PT and delayed IDT can help identify the culprit by increasing the likelihood of a positive test result.^3^ Utilizing both testing strategies were beneficial due to the variable sensitivity and specificity and preparations of medications – ceftriaxone, contrast, clindamycin are sterile and appropriate for IDT and clindamycin, amoxicillin, and naproxen were available for PT. The overlap testing options provided additional confirmatory testing for this patient.

Confirming the causative agent through objective testing is essential, relying on likelihood, frequency, or timing of administration can lead to mislabeling of drug allergies. This is problematic as it could lead to unnecessary avoidance of safer, more effective medications or inadvertently receiving the culprit medication again. In this case, clindamycin was positive on both PT and delayed IDT, which provided clarity to the trigger and single drug avoidance while allowing the patient to take all other medications in the future if needed. This case report highlights the importance of combination patch and intradermal testing to confirm medication(s) culprits in AGEP. We encourage ongoing collaboration with dermatology and allergy specialists to further best practices for drug culprit evaluation in AGEP.

## Conflicts of interest

None disclosed.

## References

[1] Parisi R., Shah H., Navarini A.A. (2023). Acute generalized exanthematous pustulosis: clinical features, differential diagnosis, and management. Am J Clin Dermatol.

[2] Vallejo-Yagüe E., Martinez-De la Torre A., Mohamad O.S., Sabu S., Burden A.M. (2022). Drug triggers and clinic of acute generalized exanthematous pustulosis (AGEP): a literature case series of 297 patients. J Clin Med.

[3] Khan D.A., Banerji A., Blumenthal K.G. (2022). Drug allergy: a 2022 practice parameter update. J Allergy Clin Immunol.

[4] Saff R.R. (2023). Skin testing as a biomarker in drug allergy. Ann Allergy Asthma Immunol.

[5] de Groot A.C. (2022). Results of patch testing in acute generalized exanthematous pustulosis (AGEP): a literature review. Contact Dermatitis.

[6] de Groot A.C. (2022). Patch testing in drug eruptions: practical aspects and literature review of eruptions and culprit drugs. Dermatitis.

[7] Broyles A.D., Banerji A., Barmettler S. (2020). Practical guidance for the evaluation and management of drug hypersensitivity: specific drugs. J Allergy Clin Immunol Pract.

[8] Barbaud A., Castagna J., Soria A. (2022). Skin tests in the work-up of cutaneous adverse drug reactions: a review and update. Contact Dermatitis.

[9] Phillips E.J., Bigliardi P., Bircher A.J. (2019). Controversies in drug allergy: testing for delayed reactions. J Allergy Clin Immunol.

